# Fruit and vegetable intake and associated factors in older adults in South Africa

**DOI:** 10.3402/gha.v5i0.18668

**Published:** 2012-11-29

**Authors:** Karl Peltzer, Nancy Phaswana-Mafuya

**Affiliations:** 1 HIV/AIDS/SIT/and TB (HAST), Human Sciences Research Council, Pretoria, South Africa; 2 Department of Psychology, University of Limpopo, Turfloop, South Africa; 3 Office of the Deputy-Vice Chancellor, Nelson Mandela Metropolitan University, Port Elizabeth, South Africa

**Keywords:** fruits, vegetables, inadequate consumption, risk factors, older adults, South Africa, WHO SAGE

## Abstract

**Background and objective:**

Numerous studies support the protective effect of high fruit and vegetable (FV) consumption on chronic disease risk, mainly against cancer and cardiovascular diseases. Compared with younger adults, older people experience additional health, social, and environmental conditions that affect dietary intake. To identify those additional dimensions and examine them in association with FV intake, data on 3,840 participants in the Study of Global Ageing and Adults Health (SAGE) in South Africa were analyzed.

**Methods:**

We conducted a national population-based cross-sectional study in 2008 with a sample of 3,840 participants, aged 50 years or older, in South Africa. The questionnaire included questions on socio-demographic characteristics, health variables, anthropometry, and blood pressure measurements. Multivariable regression analysis was performed to assess the associations between socio-demographic factors, health variables, and inadequate FV consumption.

**Results:**

Overall prevalence rates of insufficient FV intake were 68.5%, 64.8% among men and 71.4% among women, with a mean intake of 4.0 servings of FV among older adults (50 years and older). In multivariable analysis, coming from the Black African or Colored population group, lower educational level and daily tobacco use were associated with inadequate FV intake.

**Conclusions:**

The amount of fruit and vegetables (FVs) consumed by older South African participants was considerably lower than current recommendations (daily intake of at least five servings; 400 g). Public education and campaigns on adequate consumption of FVs should be promoted targeting lower educated and Black African and Colored population groups.

Worldwide, low intake of fruits and vegetables (FVs) contributes significantly to the global disease burden, including cardiovascular diseases and cancer (1). In South Africa, low fruit and vegetable (FV) intake accounted for 3.2% of total deaths and 1.1% of the 16.2 million attributable disability-adjusted life years (DALYs) in 2000 (2). The World Health Organization (WHO) (3) recommends an intake of a minimum of 400 g or five servings of FVs per day for the prevention of chronic diseases, such as heart diseases, cancer, diabetes, and obesity. The World Health Survey showed that among an adult population (18–99 years), the low FV consumption prevalence in 52 mainly low- and middle-income countries was 77.6% for men and 78.4% for women (4). In several Asian (INDEPTH, International Network for the Demographic Evaluation of Populations and Their Health, in developing countries) Health and Demographic Surveillance System sites, inadequate FV intake was found to be more than 89% in men and 96% in women in Bangladesh, 96% in men and 92% in women in Indonesia, and more than 84% in men and more than 79% in women in Thailand (5). However, little is known about the frequency, distribution, and determinants of FV consumption among older adults in South Africa. Previous studies among the adult population in South Africa in 2003 found that 72.2% of men and 66.7% of women had low FV consumption (5) and in a local study, 7% of the villagers and none of the semi-urban dwellers reported the recommended five or more FV servings per day (6). In a more recent study among adults (25–64 years) in Mozambique, less than 5% of the subjects reported an intake of five or more daily servings of FVs (7). In Iran, a middle-income country, low mean number of FV servings per day (1.76) among persons aged 60 and above were reported (8), while in some high-income countries, lower rates of FV intake was found among older adults, for example, 47% in Canada (9) and a mean consumption of 4.0 and 4.1 portions of FVs per weekday and per weekend day, respectively, in Northern Ireland (10).

Various factors have been identified to be associated with low prevalence of FV intake, including 1) Sociodemographics: older age (4, 11), male gender (11–16), lower education (5, 9, 12, 17–19), lower income (4, 9, 14, 18, 20), urban or rural residence (7, 21), and White population group or race (13); 2) Lack of social cohesion or social capital (8, 12, 22), and being single, widowed, or divorced (11, 13, 17, 20); 3) Chronic and other illness conditions: diabetes (23), hypertension (24), overweight or obese (11, 22), or not overweight (25), depressive symptoms (26); 4) Poor subjective health status (22); 5) Lifestyle factors: smokers (17, 19, 27), low physical activity (11, 19), drinking alcohol (11), and not drinking alcohol (19); and 6) Knowledge and psychosocial determinants (8, 28).

This study aims to investigate the prevalence and associated factors of low FV consumption in a national probability sample of older South Africans who participated in the Study of Global Ageing and Adults Health (SAGE) in 2008.

## Methods

### Sample and procedure

We conducted a national population-based cross-sectional study in 2008 with a sample of 3,840 participants, aged 50 years or older, in South Africa. The SAGE sample design entails a two-stage probability sample that yields national and subnational estimates to an acceptable precision at provincial level, by locality type (urban and rural), and by population group (including Black, Colored, Indian or Asian, and White). The individual response rate among those aged 50 years or older was 77%. The SAGE survey was carried out in South Africa in partnership between the WHO, the National Department of Health, and the Human Sciences Research Council (HSRC). The HSRC Research Ethics Committee and the national Department of Health approved the study.

## Measures

*Fruit and vegetable consumption* was assessed using two questions ‘How many servings of fruit do you eat on a typical day?’ and ‘How many servings of vegetables do you eat on a typical day?’ using the 24-hour dietary recall data as the gold standard. Researchers were trained to show all respondents a nutrition risk factor card that indicates both in writing and pictorially general categories, amounts, and examples of FVs in an attempt to standardize the serving size and number of servings reported. The nutrition card categorized one serving of vegetables into one of three groups: (a) one cup of raw green leafy vegetables such as spinach or salad; (b) one-half cup of other vegetables cooked or chopped raw, such as tomatoes, carrots, pumpkin, corn, Chinese cabbage, beans, or onions; and (c) one-half cup of vegetable juice. Furthermore, the nutrition card categorized one serving of fruit into one of three groups: (a) one medium-sized piece of fruit, such as an apple, banana, or orange; (b) one-half cup of cooked, chopped, or canned fruit; and (c) one-half cup of fruit juice, not artificially flavored (4). Insufficient FV consumption was defined as less than five servings of fruits and/or vegetables a day. Cronbach α for the two questions in this sample was FV 0.74.

*Blood pressure* (systolic and diastolic) was measured three times on the right arm/wrist of the seated respondent using an automated recording device. Out of three measurements, the average of the last two readings was used. In accordance with the Seventh Report of the Joint National Committee of Prevention, Detection, Evaluation, and Treatment of High Blood Pressure, individuals with systolic blood pressure ≥140 mmHg and/or diastolic blood pressure ≥90 mmHg and/or who reported the current use of antihypertensive medication were considered to be suffering from high blood pressure (29).

*Tobacco use—*Lifetime tobacco used was assessed with the question ‘Have you ever smoked tobacco or used smokeless tobacco?’ Lifetime tobacco users were asked, ‘Do you currently use (smoke, sniff, or chew) any tobacco products such as cigarettes, cigars, pipes, chewing tobacco, or snuff?’ The response options were ‘Yes, daily’, ‘Yes, but not daily’, and ‘No, Not at all’ (30).

*Alcohol use—*Lifetime alcohol use was assessed with the question ‘Have you ever consumed a drink that contains alcohol (such as beer, wine, spirits, etc.)?’ Response options were ‘Yes’ or ‘No, never’. Lifetime alcohol users were asked about current (past month) alcohol use, and current alcohol users were asked ‘During the past 7 days, how many drinks of any alcoholic beverage did you have each day?’

*Height and weight* were measured. Body mass index (BMI) was used as an indicator of obesity (≥30 kg/m^2^). BMI was calculated as weight in kg divided by height in meter squared. Overweight and/or obesity was defined as ≥25 BMI and underweight as <18.5 BMI.

*Physical activity* was measured using the General Physical Activity Questionnaire (GPAQ). This instrument gathers information on physical activity in three domains (activity at work, travel to and from places, and recreational activities), as well as time spent sitting. The questionnaire also assesses vigorous and moderate activities performed at work and for recreational activities.

Information on the number of days in a week spent on different activities and time spent in a typical day for each activity was also recorded (31). For physical activity, in addition to the total minutes of activity, the activity volume was also computed by weighing each type of activity by its energy requirement in metabolic equivalents (METs). The number of days and total physical activity MET minutes per week were used to classify respondents into three categories of low, moderate, and high level of physical activities. *High* physical activity: a person reaching any of the following criteria is classified in this category: vigorous-intensity activity on at least 3 days achieving a minimum of at least 1,500 MET (metabolic equivalent)-minutes per week odds ratios (OR); 7 or more days of any combination of walking, moderate or vigorous intensity activities achieving a minimum of at least 3,000 MET-minutes per week. *Moderate* physical activity: a person not meeting the criteria for the ‘high’ category but meeting any of the following criteria is classified in this category: 3 or more days of vigorous-intensity activity of at least 20 min per day OR; 5 or more days of moderate-intensity activity or walking of at least 30 min per day OR; 5 or more days of any combination of walking, moderate or vigorous intensity activities achieving a minimum of at least 600 MET-minutes per week. *Low* physical activity: a person not meeting any of the aforementioned criteria falls into this category. Physical inactivity was defined as those who had low levels of physical activity; moderate and high levels of physical activity were collapsed into further analysis (31). Cronbach α for the five questions on different physical activity domains was 0.77 in this sample.

*Overall self-rated health status was* based on respondents’ assessment of their current health status on a five-point scale in response to the question: ‘In general, how would you rate your health today?’ Response categories were: very good, good, moderate, bad, and very bad.

*Symptom-based depression* in the past 12 months was assessed based on the World Mental Health Survey version of the Composite International Diagnostic Interview (32). The diagnosis of depression was based on the International Classification of Diseases tenth revision (ICD-10) diagnostic criteria for research for depressive Episodes (33) and was derived from an algorithm that took into account respondents reporting symptoms of depression during the past 12 months (34).

*Social cohesion*—Social cohesion was measured with nine items, starting with the introduction ‘How often in the last 12 months have you … e.g. attended any group, club, society, union, or organizational meeting?’ Response options ranged from never=1 to daily=5. Cronbach α for this social cohesion index in this sample was 0.73.

*Religious activity*—Religious activities included frequency of attendance at religious services (measured on a six-point scale, from more than once a week to never). Responses were categorized into once a week or more=1 and once or twice a month to never=0.

*Quality of life* was assessed with the WHOQol-8 containing eight items that were empirically derived from the WHOQOL-Bref (35). The summative model was used producing an index. Cronbach α for the WHOQol-8 was 0.85 in this sample.

*Economic or wealth status*. To estimate economic or wealth status, a random-effects probit model was used to identify indicator-specific thresholds that represent the point on the wealth scale above which a household is more likely to own a particular asset than not. This enabled an estimation of an asset ladder. These estimates of thresholds, combined with actual assets observed to be owned for any given household, were used to produce an estimate of household-level wealth status. This was then used to create wealth quintiles (36).

## Data analysis

The data were entered using CSPro and analyzed using STATA Version 10. Data was weighted using post-stratified individual probability weights based on the selection probability at each stage of selection. Individual weights were post-stratified by province, sex, and age-groups according to the 2009 Medium Mid Year population estimates from Statistics South Africa. Available at: http://www.statssa.gov.za/publications/P0302/P03022009.pdf. Associations between key outcomes of insufficient FV intake and sociodemographic, social and health variables were evaluated calculating OR. Unconditional multivariable logistic regression was used for evaluation of the impact of explanatory variables for key outcome of insufficient FV intake (binary dependent variable). All variables statistically significant at the *P*<0.05 level in bivariate analyses were included in the multivariable models. In the analysis, weighted percentages are reported. The reported sample size refers to the sample that was asked the target question. Two-sided 95% confidence intervals are reported. *P* values less than or equal to 5% are used to indicate statistical significance. Both the reported 95% confidence intervals and the *P* value are adjusted for the multistage stratified cluster sample design of the study.

## Results

### Sample characteristics and prevalence rate of FV intake

The total sample included 3,840 South Africans who were 50 years or older, 44.1% men and 55.9% women. The most prevalent population group was African Black (74%); almost half (49.9%) was aged between 50 and 59 years. The educational level of most participants (71.6%) was lower than secondary school education and almost two-thirds (64.9%) lived in an urban area. A very large group (72.4%) of older adults was overweight or obese and had hypertension (77.3%), 20.4% were daily tobacco users, 4.2% had symptom-based depression, and 9.2% had diabetes. More than half (60.5%) engaged in low physical activity, 20.4% were daily tobacco, users and a small proportion (13.7%) was current alcohol user. A sizeable proportion (17.5%) rated their health status as bad or very bad and almost 10% were hungry because they could not afford to buy food every or almost every month. The overall prevalence of insufficient FV intake was 68.5% and the mean intake per day was 4.0 servings of FV, 1.8 for fruits and 2.2 for vegetables (see Table 1).

**Table 1:** Descriptive statistics of sample characteristics and prevalence rate of insufficient fruit and vegetable (FV) intake among older South Africans (male 1,638; female 2,202)

	Total sample	Male	Female	Total	Fruits	Vegetables	FV
			
	*N* (%)	Insufficient FV in %			Mean servings/day		
All	3,840	64.8	71.4	68.5	1.8 (1.4)	2.2 (1.5)	4.0 (2.5)
Age							
50–59	1,695 (49.9)	67.9	70.8	69.5	1.8 (1.3)	2.2 (1.4)	4.0 (2.5)
60–69	1,233 (30.6)	60.1	73.5	67.6	1.8 (1.3)	2.3 (1.5)	4.1 (2.6)
70 and over	912 (19.5)	63.6	69.9	67.5	1.9 (1.6)	2.2 (1.5)	4.1 (2.7)
Population group							
African Black	2,053 (74.0)	71.0	73.2	72.3	1.8 (1.4)	2.1 (1.5)	3.9 (2.6)
White	269 (9.3)	38.3	55.7	47.7	2.2 (1.3)	2.7 (1.3)	4.9 (2.4)
Colored	655 (12.8)	72.8	74.2	73.7	1.6 (0.9)	2.0 (1.1)	3.6 (1.8)
Indian or Asian	307 (3.8)	45.1	60.7	54.6	2.1 (1.4)	2.8 (2.0)	4.9 (3.1)
Marital status							
Single	512 (14.3)	65.9	75.2	72.7	1.6 (1.2)	2.1 (1.5)	3.7 (2.4)
Married	2,007 (55.9)	64.6	66.9	65.4	1.9 (1.4)	2.3 (1.4)	4.2 (2.4)
Separated/divorced	230 (5.9)	64.7	75.4	72.3	1.6 (1.3)	2.3 (1.6)	3.9 (2.5)
Widow	1,020 (23.9)	65.1	72.6	71.6	1.8 (1.5)	2.2 (1.5)	4.0 (2.9)
Educational level							
No schooling	854 (25.2)	78.2	80.0	79.4	1.6 (1.5)	1.9 (1.4)	3.5 (2.5)
Less than primary	803 (24.0)	73.1	69.2	70.8	1.7 (1.3)	2.2 (1.5)	3.9 (2.6)
Primary	779 (22.4)	68.8	68.6	68.7	1.9 (1.3)	2.2 (1.3)	4.1 (2.3)
Secondary or more	923 (28.3)	52.1	66.2	60.1	2.0 (1.4)	2.5 (1.6)	4.5 (2.6)
Wealth							
Low	1,482 (40.6)	72.8	75.8	74.5	1.9 (1.1)	2.2 (1.3)	4.1 (2.1)
Medium	731 (18.2)	67.7	70.3	69.5	1.7 (1.4)	2.1 (1.4)	3.8 (2.5)
High	1,608 (41.2)	56.5	67.1	61.9	2.0 (1.7)	2.6 (1.9)	4.6 (3.4)
Geolocality							
Rural	1,276 (35.1)	73.7	76.2	75.1	1.7 (1.6)	2.1 (1.7)	3.8 (3.0)
Urban	2,561 (64.9)	60.1	68.7	64.9	1.9 (1.2)	2.3 (1.3)	4.2 (2.3)
Other conditions							
Hypertension	2,842 (77.3)	65.3	68.7	67.3	1.8 (1.4)	2.3 (1.5)	4.1 (2.6)
Symptom-based depression	161 (4.2)	80.5	70.6	74.7	1.6 (1.4)	2.0 (1.3)	3.6 (2.4)
Diabetes	360 (9.2)	57.5	65.5	62.8	1.9 (1.4)	2.5 (1.7)	4.4 (2.8)
Overweight (BMI ≥25)	2,505 (72.4)	59.5	68.8	65.0	1.9 (1.4)	2.3 (1.5)	4.2 (2.6)
Eat less because of no food							
Every or almost every month	291 (9.9)	69.8	75.8	73.6	1.6 (1.9)	1.9 (1.5)	3.6 (2.8)
Some months or only 1 or 2 months	637 (19.2	54.7	61.0	58.5	2.1 (1.6)	2.6 (1.9)	4.7 (3.3)
Never	2,699 (70.9)	64.4	72.5	68.8	1.8 (1.2)	2.2 (1.3)	3.9 (2.2)
Hungry because could not afford							
Every or almost every month	312 (9.6)	72.7	76.8	75.3	1.6 (1.9)	1.9 (1.4)	3.5 (2.8)
Some months or only 1 or 2 months	708 (21.8)	54.7	61.7	59.0	2.2 (1.6)	2.6 (2.0)	4.8 (3.4)
Never	3,630 (68.5)	64.5	72.4	68.7	1.8 (1.2)	2.2 (1.3)	3.9 (2.2)
Daily tobacco use	810 (20.4)	74.4	76.8	75.6	1.6 (1.4)	1.9 (1.2)	3.5 (2.2)
Alcohol use (past month)	557 (13.7)	71.4	77.6	73.5	1.6 (1.4)	1.9 (1.2)	3.5 (2.4)
Physical inactivity	2,455 (60.5)	64.2	70.6	67.9	1.9 (1.5)	2.3 (1.7)	4.2 (2.9)
Subjective health status							
Very/good	1,469 (37.9)	61.2	69.8	65.6	1.9 (1.1)	2.2 (1.3)	4.1 (2.1)
Moderate	1,681 (44.9)	70.3	75.2	73.2	1.7 (1.4)	2.1 (1.4)	3.8 (2.5)
Bad/very bad	617 (17.5)	58.2	62.3	60.5	2.0 (1.7)	2.6 (1.9)	4.6 (3.4)
Religious involvement	1,596 (52.9)	60.7	68.1	65.1	1.9 (1.4)	2.4 (1.6)	4.3 (2.8)
Social cohesion index (range 9–72); M (SD)=	22.1 (6.5)	22.7 (7.0)	21.8 (6.7)	22.1 (6.7)			
Quality of life (range 0–100)	47.1 (12.5)	45.5 (12.6)	47.2 (11.9)	46.2 (12.3)			

### Predictors of insufficient FV intake

In univariate analysis, male gender, coming from the Black African or Colored population group, low educational level, not being overweight, daily tobacco use, lack of religious involvement, and lower quality of life were associated with inadequate FV intake, while in multivariable analysis, coming from the Black African or Colored population group, lower educational level, and daily tobacco use were associated with inadequate FV intake (see Table 2).

**Table 2:** Univariate and multivariable analyses of association of demographic, social, and health factors with insufficient fruit and vegetable (FV) intake in older South Africans

	Unadjusted odds ratios (95% CI)	Adjusted odds ratios (95% CI)
Gender		
Female	1.00	1.00
Male	0.74 (0.61–0.89)**	0.82 (0.65–1.04)
Age		
50–59	1.00	1.00
60–69	0.92 (0.71–1.18)	1.06 (0.82–1.38)
70 and over	0.91 (0.71–1.17)	0.95 (0.69–1.32)
Population group		
Black African	1.00	1.00
White	0.35 (0.15–0.80)*	0.46 (0.22–0.97)*
Colored	1.07 (0.64–1.80)	1.18 (0.72–1.92)
Indian or Asian	0.46 (0.25–0.83)*	0.53 (0.28–0.98)*
Marital status		
Single	1.00	–
Married	0.71 (0.39–1.29)	–
Separated/divorced	0.98 (0.52–1.84)	–
Widow	0.94 (0.49–1.81)	–
Educational level		
No schooling	1.00	1.00
Less than primary	0.93 (0.61–1.40)	0.64 (0.40–1.04)
Primary	0.84 (0.54–1.31)	0.56 (0.31–1.01)
Secondary or more	0.57 (0.38–0.86)**	0.55 (0.35–0.86)*
Wealth		
Low	1.00	–
Medium	0.78 (0.55–1.10)	–
High	0.56 (0.30–1.02)	–
Geolocality		
Rural	1.00	–
Urban	0.61 (0.26–1.43)	–
Other conditions		
Hypertension (examined and self-report)	0.87 (0.58–1.29)	–
Diabetes	0.80 (0.52–1.22)	–
Overweight	0.66 (0.49–0.87)**	0.90 (0.65–1.26)
Symptom based depression	1.45 (0.64–3.29)	–
Eat less because of no food		
Every or almost every month	1.00	–
Some months or only 1 or 2 months	0.47 (0.15–1.48)	–
Never	0.72 (0.35–1.47)	–
Hungry because could not afford		
Every or almost every month	1.00	–
Some months or only 1 or 2 months	0.51 (0.16–1.65)	–
Never	0.79 (0.41–1.53)	–
Daily tobacco use	1.65 (1.13–2.40)**	1.67 (1.07–2.59)*
Alcohol use (past month)	1.40 (0.82–2.40)	–
Physical inactivity	0.94 (0.57–1.53)	–
Subjective health status		
Very/good	1.00	–
Moderate	1.43 (0.89–2.32)	–
Bad/very bad	0.80 (0.41–1.56)	–
Social cohesion index	1.00 (0.97–1.03)	–
Religious involvement	0.71 (0.53–0.91)*	0.95 (0.66–1.36)
Quality of life	0.95 (0.91–1.00)*	0.98 (0.95–1.01)

** *P*<0.01;

* *P*<0.5.

## Discussion

This is one of the first studies among older adults in Africa showing significant prevalence rates of insufficient FV intake (68.5%; male 64.8%, female 71.4%), with a mean intake of 4.0 servings of FVs in South Africa. These rates seem to be similar to a previous survey among the adult population in South Africa (4) and in a study among older adults in Northern Ireland (10), but the prevalence rate of insufficient FV intake was lower in this study than among elderly populations in several Asian countries (5), Mozambique (7), and Iran (8) and it was higher than in Canada (9). Unlike other studies (11–16), the prevalence of low FV intake was higher (although not significantly) among female than male older adults in this study. However, this was also found in an adult survey in South Africa (4). We speculate that this might be due to cultural differences among countries.

The study further found that regarding sociodemographics, coming from the Black African or Colored population group and lower educational level, as found in many studies (5, 9, 12, 17–19), were in multivariable analysis associated with inadequate FV intake. Education, in this case secondary or post secondary education, was found to be associated with a significant rise in the proportion of the South African elderly who reported adequate intake of FVs. Some studies found that knowledge regarding FVs plays an important role in adequate FV intake (8, 11, 37). In future studies, it would be important to assess the relationship between education levels of older adults and their knowledge about FVs so as to ascertain if knowledge about FVs is the main factor determining adequate FV consumption (11). The finding that the Black African and Colored population group were the highest in inadequate FV intake is not compliant with previous studies (13). Therefore, increased efforts should be made to target the Black African and Colored population with FV intake messages. Unlike in some other studies (4, 7, 9, 11, 14, 18, 20), this study did not find any effect of age, income, and geolocality on inadequate FV intake. In this study, we found that the poorest wealth quintile had a higher prevalence of FV consumption compared to those from the richest wealth quintile. This finding may not provide support for studies (38, 39) that indicate that FV prices are a barrier to consumption of low-income consumers. As a result, developing policies and programs were needed to make FV more affordable for the poor. However, further studies are needed to explore the relationship between income, price, consumer preferences, and healthy diet (FV intake) (39, 40).

In terms of health variables, in agreement with other studies (17, 19, 27), this study found that tobacco use was associated with inadequate FV intake. Unlike some other studies (11, 19, 22–26), this study did not find low physical activity, alcohol use, weight status, diabetes, hypertension, depressive symptoms, and poor subjective health status to be associated with inadequate FV intake. Furthermore, unlike in other studies, lack of social cohesion or social capital and being single, widowed, or divorced (8, 11–13, 17, 22) were not associated with FV intake in this study.

## Limitations of the study

This study had several limitations. First, the self-report of health variables such as FV intake, physical activity, tobacco or alcohol use should be interpreted with caution; it is possible that measurement errors occurred (40, 41). The two FV questions depend on memory, therefore, there is the possibility of recall bias (4). However, this bias is minimized since the questions refer to a very short time period. One main limitation is that this method may not provide reliable estimates of the usual intake of participants. The self-reported assessment of physical activity remains the most feasible and affordable instrument for global surveillance. However, objective population measures of physical activity, such as pedometers (42) or accelerometers (43), may be beneficial to determine if differences between groups revealed in the present study represent true differences in physical activity behavior. The study did not validate the measurement of dietary intake of FV and physical activity, which are highly susceptible to respondent bias in the form of under or over reporting. Many studies in the area of nutritional epidemiology have established the need to integrate validation of measurements of dietary intake and physical activity into the study design. Without such an inbuilt component, the internal validity of the measurements are compromised. Second, information on knowledge, psychosocial determinants (habit, motivation, goals, beliefs about capabilities, skills, taste, attitudes, and self-efficacy) (8, 28) and environmental factors such as local availability (20) were not collected and should be included in future studies. Further, seasonal differences in availability of FVs may have influenced the consumption patterns and should be assessed in future studies (11). Finally, this study was based on data collected in a cross-sectional survey. We cannot, therefore, ascribe causality to any of the associated factors in the study.

In conclusion, the amount of FVs consumed by older South African participants were far below the level of current recommendations (daily intake of at least five servings; 400 g). Public education and campaigns on adequate consumption of FVs should be promoted targeting more lower educated and Black African population groups.
